# An Essay on Remittent and Intermittent Diseases, &c.

**Published:** 1829-04-01

**Authors:** 


### AN ESSAY ON REMITTENT AND INTERMITTENT DISEASES, INCLUDING MARSH-FEVER, NEURALGIA, &c. &c. By *John Macculloch*, M.D. F.R.S. &c. Two Volumes, Octavo, 1827-8.

[Art. IV. (and last) TREATMENT OF NEURALGIA.]

WE are now enabled to complete our analytical review of Dr. Macculloch's interesting and valuable volumes—and we do not regret or apologise for the great space which we have occupied in this analysis. We are convinced that we have, through this medium, disseminated more original and important matter, and that to a greater extent, than has ever before been done through the vehicle of a review. We are confirmed in this opinion by the numerous applications which we have, from time to time, received, soliciting a further prosecution of the analytical delineation of our author's volumes. We now hasten to put the finishing hand to our task.

### CHAP. X.—RHEUMATISM OF THE EYE—OR, NEURALGIA OPHTHALMICA.

Dr. M. assures us that the doctrine of the malarious nature of neuralgic ophthalmia was entertained by him a great number of years ago—and has since been annually confirmed by extensive personal observation. Dr. M. remarks that, whenever medical practitioners shall pay minute attention to the distinction between this disease and common ophthalmia, they will find plenty of examples. The loss of sight is not an uncommon consequence of want of discrimination in such cases.

"In some places, this ophthalmia is arranged by Sauvages with his Migraine or Hemicrania, under the term 'migraine des yeux,' and in others under other titles: proving his want of correct notions respecting it: while Cullen does not take the slightest notice of it in his very meagre and superficial description of the general disorder. The former remarks that it produces inflammation in the globe of the eye, terminating in a confusion of the humours and in suppuration; unaware apparently of the milder cases, yet, in another place, noticing its tendency to return in the second eye after destroying the first. In St. Yves and Maître Jean, some cases of it are described by the term *amaurosis;* that expression apparently meaning the destruction of the humours: while it is remarked that it endures for months or years, that it is attended by fever, hemicrania or clavus, want of sleep, and giddiness, and that it attacks one eye after the other: while the former oculist, adopting the usual resource which ignorance has resorted to in so many more of these disorders, proposes to prevent this second attack by extirpating the first eye. I did not say too much when I said that had the surgical sect believed the sciatica to be Neuralgia, and dared to extirpate the sciatic nerve, they would equally have had recourse to their panacea, the knife.

"Among many casual notices of this peculiar ophthalmia, and unsatisfactory as casual, I must however distinguish the essay of Wardrop; the first, I believe, through which the attention of physicians was fairly called to it. To praise that essay for the accuracy of its description of a much neglected disease, is but to agree with all who have read it: yet I should be negligent of my duty did I not remark, that however perfectly my able friend has seen and discriminated this variety, his account is limited to the severer cases, and that he has not appeared to be aware, in his essay, whatever may be the case otherwise, either of the slighter and less marked, or even of the chronic, varieties, or of the extreme prevalence of this peculiar species; and that, in consequence, there is almost as much error prevailing, in practice, respecting it, even under the name which he has adopted, by those who have had the advantage of his experience, as there was before. Thus also I perceive no notice of its properly intermittent and alternating characters; while with respect to other portions of the description, such as the general fever, the bilious symptoms, the decided neuralgic and periodical pain, to which I may also add the utility of bark, I should desire no other evidence to prove that it demands the term which I desire to apply to it, and that it is in reality a mode of Neuralgia. I shall however be able to produce much further evidence of its connection both with that disease and with intermittent: and if I were inclined to express any surprise that so acute and experienced an observer had not formed the conclusion to the very verge of which he has approached, I should suppress that by recollecting, that in this as in every other disease which I have here decribed under this leading character, the foundation and cause of all the error must be sought in the want of a correct and broad view of the fundamental disease itself: Neuralgia." 252.

Dr. M. acknowledges that, on farther research, he has found notices of the connexion between ophthalmia and intermittent, in the writings of Morton, Strack, and Monfalcon —the latter remarking that this kind of inflammation is very common in the malarious districts of France, and is very apt to terminate in opacities. Like all the neuralgic diseases, this one sometimes occurs under a periodical character—at others, it is irregular. But the same may be said of rheumatism of the face, of which it is a near relative.

"It is observed, and perhaps very commonly with truth, that this ophthalmia is produced by exposure to cold winds, very often by partial cold, and very particularly, as it is thought, by a sudden impulse of the east wind on the eye, or face. The popular term in this case is, a 'blight;' while as it is not unusual for the east wind to be especially attended by dust, this is often esteemed the exciting cause, and is as often vainly sought after; the patient being misled by the well-known sensation which follows the enlargement of the small vessels.

"Now, so far from this view of the cause being averse to the opinion of its belonging to the class of intermittent and neuralgic disorders, cold so applied is precisely one of the causes which produces these also, just as it excites the rheumatism of the face; adding a proof, such as it may be thought, respecting the true nature of that disease as well as of the ophthalmia in question. An average of cases will show that the rheumatic ophthalmia is much more common in spring and during east winds than at any other time, and the very vulgar themselves are indeed convinced of this as to ophthalmia generally: while it will I believe be found, that nine cases of ten, or indeed far more, if not even all of the ophthalmias thus occurring, are this very disease. And I formerly showed, while I have attempted to explain the cause, that such east winds, at that season of the year, do produce intermittents as well as Neuralgia; so that as far as cause is concerned, the whole of these disorders unite under one general head, instead of being separated by differences of cause: while it is still easy to see how the local action of cold on the eye or face, might determine the local disease especially; the wind thus acting by a double power." 256.

The above reasoning is equally ingenious and just—and it will be found, on accurate observation, that this disease abounds most in those seasons or years in which marsh-fever rages most. It is completely proved by the geographical bearings of this ophthalmia, that malaria is at least its principal cause. It is endemic on all the coasts of the Mediterranean where fevers prevail, occurring very remarkably, at the same season, or in the pestilential months of Summer and Autumn. It prevails along the marshy coasts of Barbary, during four months of the year—while at Tripoli few escape it. It is common at Rome, Naples, and other parts of Italy, where malaria is acknowledged to be predominant. In Spain, this ophthalmia is extremely prevalent on the maritime coasts that are subject to fever—especially at Valencia, Albatera, and Clivillente. The following is the graphic description of the disease itself, as drawn up by our very intelligent author.

"There is a peculiarity in the aspect of the inflammation itself, far easier to recognise than to describe, and by which alone it is generally distinguishable, even at a distance, and on a mere glance, to those who have acquired that experience which in other cases is called the *tactus eruditus*. I have sought in vain for expressions to say fully what this is; but I believe it to be as useless as difficult, since, however accurate they might appear to those who already know this inflammation by sight, they would not teach others to know it, inasmuch as no visible object can be justly described to the previously ignorant; while such a description would be useless to those who are already experienced in this ophthalmia. The more obvious character, however, is a dull, rather than a lively red colour, not unfrequently attended by a tinge of yellow; the cause of which is especially visible in the sound eye when but one is inflamed, and the source of which must now also be obvious, particularly in autumnal cases. This inflammation occupies the whole conjunctiva, even to the verge of the cornea; and while the redness is rather produced by the minutest branches of the arteries than the larger ones, the general aspect is almost that of an additional coat of red cloth in the severer cases, sometimes attaining a higher level than that of the cornea.

"In severity, however, it differs exceedingly, from a mere general, and somewhat pale, redness of the conjunctiva, to that violent inflammation just noticed. Here, it is apt to resemble the celebrated contagious and purulent ophthalmia; but it can nevertheless be distinguished by attending to its progress and to the collateral symptoms, while it never, as far as I know it, suppurates on the surface, like that disease. This is a part of its history however on which I must yet speak with some hesitation; as, after many years of observation, whence I concluded that it never did suppurate, my opinions have been recently shaken by one or two cases, though I had not the opportunity that was necessary for satisfying myself as to the real nature of the disease in these. Whenever it shall, as a separate disease, have received from physicians the further attention which it requires, this, and some other circumstances which I cannot now well elucidate, will be better understood; while I shall gladly avail myself of such information; though it will be necessary that this disorder shall be truly discriminated for this purpose, lest we return into worse confusion than that which I am attempting to rectify." 261.

Such is the general and obvious character; but there are one or two remarkable circumstances that yet deserve remark. It is often unattended by any pain in the eye itself—especially where it is of long standing and not very severe. There are cases, however, where the pain and irritation are as great as in purulent ophthalmia. This is a peculiarly obstinate and untractable disease—lasting, for months, as a mere deformity, and with little suffering, resisting every means of relief—and being generally aggravated by depletive measures.

Mr. Wardrop has remarked a peculiar sense of dryness in the eye at the commencement—followed ultimately by a copious lachrymation. The latter symptom Dr. M. can confirm by ample observation. He cannot so speak of the first.

"When there is no pain in the ball of the eye, it would seem that the conjunctiva alone is affected; while, when irritability to light attends, we must suppose that the neighbouring vessels, and nerves, within the eye, are in that state, be it from sympathy or extension, which so often occurs in the rheumatism of the face, and in common Neuralgia, where, added to the decided inflammation and pain, there is an excitement, a tenderness, or an irritability in the adjoining parts. It is not necessary that the eyelids should be affected, or that the inflammation of the conjunctiva of the eye should extend over that of the eyelid; though this happens in the severer cases, and, as it would seem, rather in the acute than the chronic ones. It is a fortunate circumstance, that this inflammation is so much and so often resisted by the transparent cornea, as is the fact also in some other ophthalmias; but abundant instances of this do nevertheless occur. Rigidly speaking, and in the severer cases, the cornea becomes dull; and if this opacity proceeds, it at length forms a cloud or a spot which diffuses itself over the whole eye, while it is more condensed in the centre. Fortunately, even when very considerable, this commonly disappears, under proper treatment of the general disease, and even within a day or two; while I have seen it return many times, under different relapses and in successive seasons, without any more permanent effects. In such cases also, it will sometimes be found, by means of a lens, that there is an ulterior disorder of the cornea, resembling very superficial ulceration; equally disappearing, and without bad consequences, with the general inflammation. Far more rarely does it affect the iris; but cases even happen, as I shall soon show, where that membrane alone is the seat of the disease; the neuralgic affection producing here a rheumatism of the iris; to adopt the common phraseology." 264.

The neuralgic ophthalmia sometimes attacks suddenly, and arrives at its full degree of intensity in a few hours;—but it is often *preceded* by an intermittent or remittent febrile state, which, however, is too generally overlooked. Some symptoms of this kind will almost always be detected *during* the attack, a fact which we can substantiate by personal observation. Sometimes this ophthalmia is the sequela of a neuralgic pain in the face, as in the eye-brow, temple, or lower jaw—or even in remote parts of the body. All these circumstances mark distinctly its connexion with intermittent and neuralgic affections generally. Great modifications will be produced in the characters of this complaint, according as it is in an acute or a chronic form. The following circumstances will, however, be detected by all careful observers.

"A watchful physician will rarely fail to perceive that physiognomical mark of a cold stage at some period of the day, which I have so often pointed out; as the fever of this disorder is generally, but not invariably, a quotidian; while in many cases, that stage, and even a hot fit also, are distinctly marked. This is true even of the slightest varieties and of the most chronic or most habitual and repeated ones: while in the severer, acute, disease, the fever is strongly marked as a remitting, or even as a continuous one; as continuous at least as in simple remittent; though, under types more distant than quotidian, I have met few of a severe character; those of a tertian form which have occurred to me having been most commonly mild, or else chronic cases.

"Such a febrile state is often, as usual, paroxysmal while the inflammation is permanent; but this is no cause for surprise, as the same happens in the rheumatism of the face, in that of the intercostal muscles, and in other analogous affections, and also not unfrequently in the purer Neuralgiæ, as in sciatica. Supposing this febrile state to be present or not, or to be more or less distinct, there is frequently a separate neuralgic pain accompanying the inflammation, throughout the disease, or, occasionally, for some days only; being the hemicrania, or the pain in the temple, or in the eyebrow, which I formerly noticed as sometimes preceding the attack; and being sometimes an extremely severe Neuralgia. This is the symptom which forms that criterion for the disease which ought never to be mistaken, though in reality rarely attended to; and it is so marked and so discriminating, that to pass it without notice, or, when present, to treat the disease as common ophthalmia, is unpardonable in even the most mechanical practitioner." 269.

In the greater number of cases, only one eye is affected, though sometimes both may be the seat of the complaint—at least in succession or alternately. This is a highly discriminating feature in the complaint, and should always excite suspicion as to the nature of the ophthalmia. Another remarkable peculiarity is the disposition to migration or metastasis. When this occurs, the original inflammation, however severe, sometimes disappears entirely—even within a few hours, so that its former existence could scarcely be suspected—the new one attaining to its almost violence in a time as short—often to disappear in its turn. Our author naturally expresses his surprise that so extraordinary a fact as this should not have, long ago, attracted suspicion, since nothing analogous to it occurs in other diseases, with the exception of gout. Like other neuralgiæ and intermittents, it may be limited to one attack—or, having occurred once, it may be liable to relapse repeatedly. Like all other diseases, it often disappears spontaneously, while the remedies gain a credit to which they are not entitled.

"As to the theory of neuralgic ophthalmia, if it is not very evident, it is at least as intelligible as that of any other form of neuralgic inflammation. Of the true, the ultimate theory, of any inflammation, we know absolutely nothing; since, after all that has been written on this subject, we have but so many words; one term substituted for another. If all that we can know as yet of the cause of neuralgic inflammation is no better, it is at least not worse; while we are in no want of analogies, or the difficulty, such as it is, is countenanced by parallel difficulties." 273.

The author next proceeds to the narrative of two or three cases—because such narratives often excite an attention that would not otherwise be commanded by the most laboured general description.

"In the first case that I shall notice, which was not under my own care, but under that of a medical friend particularly interested in the result, the original disorder or attack was a periodical and daily rheumatism in the neck, remarkably well defined. After this had lasted a week, there occurred suddenly a pain in the eye, with inflammation of a very violent character. I entertain no doubt that the intermittent form remained either in the febrile symptoms or in the pain about the eye: the violence and acuteness of this being a very discriminating mark, as it does not happen in any other ophthalmia. But as this physician had never considered the rheumatism in question as a disorder belonging to intermittent or Neuralgia, he had paid no attention to the symptoms, and was therefore unable to describe the case more minutely. Far less had he ever considered any ophthalmia to be a disease of this nature; and the patient was therefore treated in the usual manner, with the unfortunate termination in blindness, from the formation of a pustule in the cornea. I have given this case as I received it from the physician himself, so that others may judge; while the suddenness and violence of the attack of inflammation, the accompanying severe pain, and the previous periodical rheumatism, leave no doubt in my own mind respecting the nature of the disease." 275.

In the following case our author attended with a most learned and talented physician—not a routine practitioner, and who was yet misled—shewing the necessity for a new investigation of this class of complaints.

*Case* 2. "In this instance, and where the personal interest was as great, the patient was suddenly attacked in the evening with an inflammation of one eye, which ceased by the following morning. On the next evening, there was no inflammation; but it returned on the alternate one, and in the other eye, terminating similarly on the following morning. As I chanced to reside in the house, I could perceive and point out the tertian cold stage; this being evidently a tertian intermittent, or rather, that disorder doubled, (not double tertian,) inasmuch as the succeeding fits were different. Nothing was done; as it was wished to watch the natural progress of the disorder, which, after lasting thus about ten days, or displaying six different alternations of this nature, became a decided double tertian; the inflammation returning every evening in the alternate eyes, to terminate in the morning. And in this instance, the neuralgic intermittent pain occurred in each eyebrow alternately, accompanying the inflammation; so as to produce a case as strongly marked as is easily conceived. I shall only add that it was afterwards cured by bark; but that I did not even then succeed in producing a free assent to opinions which, probably, I might even now have kept to myself, for all the impression they are likely to make for these twenty years to come; when those who have been most active in opposition, will be among the first to recollect that all this was long ago their own opinions." 280.

*Case* 3. In the last case which our author notices, the patient had been for some time afflicted with a general or diffused periodical rheumatism, followed at length by inflammation of both eyes, the original disease continuing. The disease was of such long standing that a cure was hopeless. Both pupils were so contracted that a pin could with difficulty have passed through one, the other being absolutely closed.

"By the patient's account, he had been seized with occasional fits of blindness during the progress of the disease, arising doubtless from the contraction of the pupil, while I have as little doubt that the Iris was affected by the neuralgic inflammation. I could not obtain a more minute account of the case, as he was a man in low life, and had no medical attendant; but enough remained to prove that the judgment I had formed was correct. For, at this time, though one eye seemed hopelessly obstructed, the other was occasionally of use; while the patient observed, and without inquiry, or leading question, that whenever the general fits of rheumatism in the limbs came on, the eye became blind, from the closing of the pupil, recovering again when those ceased. I need only add, that as the disease had at this time lasted many years, the fits were no longer as regular as they had originally been, as happens in all chronic intermittent disorders; and as the contraction of the pupil accompanied them then accurately, it is probable this had done so from the commencement, though the exact particulars had been forgotten." 281.

Dr. M. suggests, that amaurosis is sometimes dependent on a neuralgic affection, and informs us that, while his work was in the press, two well marked cases of amaurosis of one eye, produced very pointedly, and within a few weeks, by a neuralgia occupying the external part of the orbit. The gradual paralysis of the nerve, and the total absence of all other affection of the head, or of the corresponding eye, offering evidence as clear as could be desired, of the real source of the disease, and of the truth of the above conjectures."

"I may here introduce a fact which appears to me to bear on this question, and on the original one, viz. the power in this respect, of the inflammatory diseases; but of the value of which I shall suffer others to judge. This fact is, that in the Mediterranean, and in the same districts where that ophthalmia which I suppose to be the disorder under review is common, *nyctalopia*, as it is there improperly called, or in reality, the loss of vision after sunset, is a very common affection: while I need not remark that this is, in fact, a modified amaurosis, or a partially, or moderately paralytic affection of the retina or nerve." 289.

After a philippic against the sub-divisions of the medical art into oculists, aurists, dentists, &c. and still more against spine, liver, and stomach doctors, our author enters on a consideration of the treatment of rheumatic ophthalmia. There can be no doubt that, while this inflammation was or is confounded with common ophthalmia, and the usual depletory measures employed, the practice will be not only unsuccessful, but even injurious. The fundamental error consists in looking at the disease as a purely local inflammation, and over-looking

"the constitutional affection which belongs to every neuralgia." Mr. Wardrop is acknowledged to have perceived the difference between the rheumatic and the common ophthalmia, though not the true nature or cause of the former. Mr. W. varied the treatment, and with success. The chronic cases of ophthalmia too, which form the overwhelming majority, are still confounded with common inflammation, and consequently maltreated. The bark has been used either empirically, or at the end of treatment conducted on opposite principles.

Dr. M. has already said, that in all intermittent and neuralgic diseases, the evacuating and debilitating system is pernicious—more especially blood-letting, both general and local. He does not deny, however, that in some cases of intermittent and remittent fevers, moderate depletion, at the beginning, may be useful.

"Thus it is in this ophthalmia, when violent, and particularly on the first attack; since the effect may often be to reduce the local disease which threatens local injury; while, though that remedy be really pernicious as it regards the constitutional affection, inasmuch as it commonly renders that more obstinate, the evil from this cause would be as nothing compared to the possibly impending local evil. It is plain therefore that I do not absolutely exclude blood-letting, both general and local, in this ophthalmia, at least when recent and severe; yet I think it highly essential that the reasons for permitting its use should be duly understood, as I trust they will now be by reverting to what was formerly said on this subject." 298.

The disease, in short, is not to be considered as a purely local inflammation, but as a peculiar disorder connected with and dependent on a constitutional cause, which cause is inconsistent with the depleting system, except in rare cases. In the chronic cases of this ophthalmia, the evacuating system is positively injurious—and the consequences sometimes most serious. Low diet and abstinence from wine are considered by our author among the *lædentia* in this class of complaints. The following case will prove illustrative of this and of several other points under discussion.

"The person in question, an artisan under the patronage and care of an opulent family delighting in physic, was seized with the common neuralgia of the face, occasionally in its more ordinary form, and at other times under that of toothach. I was permitted to cure this by means of arsenic; but after a short time it returned in the temple, and was then followed by a tolerably severe ophthalmia, affecting the conjunctiva of the neighbouring eye, and also attacking the iris. Nothing could be better marked than the disorder; as it was attended with a distinct intermittent and quotidian cold stage, and as the neuralgia of the temple was equally regular; while the contraction of the iris was also as periodical, occurring once a day, and lasting a determinate number of hours.

"I attempted of course to explain my views of the character of the disease, while I proposed the method of cure; and with exactly the same success which I have generally had, as well with patients as with my brethren of the profession, for these twenty years and much more; at the manner of which I can now but smile, while I regret the price at which the unfortunate patients have so often purchased this imaginary triumph.

"The patient was therefore sent to an oculist, at that time of high reputation; it having been concluded, as it is still, that neither physician nor surgeon could possibly understand a disease of the eye like the man of experience; such are the ideas attached by the vulgar to a word, which, if their meaning was the true definition of that term, would make the oldest nurse, or the empiric who sees a hundred patients in a day, the best physician; just as he who has manufactured the most tons of Glauber salt and calomel in his life-time, is the most philosophical chemist.

"If out of humanity to the unfortunate patient, I attempted to explain the case to the oculist, the suggestion was received just as I expected; and, from that time,

I could but watch, for instruction, the progress of the case. The first effect of local blood-letting, blistering, and topical applications, was a great increase of the inflammation; and as the same means were continued and repeated, the disorder became daily more severe; while, the Neuralgia also increasing in severity and extent, and the intermittent becoming much more strongly marked, it was declared that there was a flow of blood to the head; and so forth. General blood-letting from a vein, together with that from the temporal artery, was therefore adopted and repeated; while after a certain progress in this practice, aided by more topical remedies, more purging, and more low diet, the patient became so ill that he could no longer attend the oculist, and was therefore sent to an hospital. These operations occupied about two months; and if I was, after this, cut off from as frequent a sight of the patient as formerly, I was easily able to ascertain, before this imprisonment, that he was labouring under an inveterate quotidian intermittent, with a Neuralgia that scarcely left any repose, extreme debility with various nervous affections, and a partial fatuity; all of them the effects which I had gradually foretold to his patrons, as any one may foretel them under such practice; while the inflammation was such as apparently to extend to the bottom of the eye, from the excessive and constant pain, and while total blindness on that side had also resulted from the complete closing of the iris.

"In the hospital, all this, in the usual way, justified more bleeding and more of every thing which had already proved so injurious; while the disease persevered without a single feature of alteration, except for the worse, during nearly three months, when the gradually increasing fatuity became a mania, and the patient attempted to destroy himself by cutting his throat. The attempt was however unsuccessful; and after the wound was healed, he was sent home, to be transferred to a lunatic asylum, during which interval, I was enabled for a week or more, to see him daily. He was then in a state of melancholy fatuity, rather than of proper mania; while the inflammation continued, but in a comparatively mild state, with occasional headach, of apparently of great severity and still periodical, though the state of the intellect prevented any very accurate examination. What was done in the lunatic hospital, I could never discover; but in about two months he died, and, as I understood through his wife, with the eye still in the same condition." 308.

The above case is extremely interesting; and our author avers, that every symptom, as well as the general progress, is that which occurs, in a greater or less degree, not only in this ophthalmia, but in every anomalous intermittent and neuralgia, "wherever the evacuant practice has been pursued."

"Of the topical applications I must observe, that there are even acute cases of this disorder, sufficiently teasing to the patient, and even alarming to timid ones, where the mere local use of stimulants does alone remove the disorder; the constitutional affection in such instances being perhaps trifling, or even, it may be supposed, nothing; or else disappearing spontaneously, or from slender changes of circumstances, as intermittents themselves, equally slight, so often do. In such cases, I know not that any thing is more efficacious than hot water, as hot as it can be endured; while in the chronic relapsing attacks it is often sufficient alone to the cure. In all these cases, acute as well as chronic, persistence is most necessary: but in saying this, I must also remark on a mistaken and injurious practice, not very rare, namely, that of applying ice; while I ought to say, generally, that cold washes of all kinds are either useless or mischievous. In the chronic cases also, especially, very strong metallic solutions, such for example as sulphate of zinc in the proportion of ten grains to the ounce of water, often remove the inflammation; while this particular class of remedies also dissipates the opacities of the cornea, unless caused by pustule or ulceration, or unless very dense, from repeated attacks. No incurable opacity from mere inflammation in this disease, ought in fact to exist: and when it is not prevented or cured, there has been neglect somewhere. Of other applications, opium, both within and without the eye, is often also useful: but let me remark here, that its chief, or almost sole value, is when applied on the subsiding of the paroxysm, or during that remission which can always, with care, be discovered:

diminishing thus, as in all the neuralgic affections of tender parts, that soreness, or pain, or uneasiness, which persists after the proper paroxysmatic attack has passed away. Thus is also the internal use of opium useful in the same circumstances. As far as this substance is of use as an application in the chronic cases, it must, I think, be classed with the stimulant remedies already mentioned.

"Lastly, in all the modes of this disease, that is, in the chronic ones at all times, and in the acute, whenever the febrile state permits, or when such evacuation as may be judged necessary has been premised, the remedies are those of intermittents and neuralgia; namely, Bark, and the tonics, under all those regulations which I need not again discuss; though I ought to remark, that in numerous cases of the long continued and relapsing disease, and in many indeed of the acuter or more severe ones, and even when of some standing, I have found both arsenic and bark eminently successful without any other aids, while rarely failing to cure a new case within even a few days. As usual in all other cases of Neuralgia under every variety, the inflammation has been more tantalizing, and the remedies less actively efficacious, as it has been of longer standing and more subjected to a previous course of maltreatment; though I can scarcely, with any effort, recall a case to mind, of whatever character, which was not cured, when the patient's confidence corresponded to my own. These, in reality, are the true remedies of this ophthalmia; a fact which if it had been always known, would have saved thousands from blindness as from suffering; and not only so, but from broken constitutions or even worse evils; since the long persistence of this disorder produces the same effects as the similar duration of any intermittent or any Neuralgia." 318.

To the above remedies must be added good diet and wine, by which alone the disease may sometimes be cured, when protracted by an opposite system. For more minute observations on the remedies, the reader is referred to the general discussion on the treatment of neuralgia.

### Chap. XI.—On the Connexion between Neuralgia and Intermittent.

If our author has shewn that all the diseases treated of in this Essay do occasionally or often arise from malaria—not, of course, excluding the occasional action of other causes, as cold, &c. it forms a strong proof of a community in the nature of these diseases. The following passage offers an example of this family connexion.

"In this case, the situation was so decidedly subject to Malaria, that scarcely an individual, out of many different families which had resided in it, had escaped intermittent at some period of their stay. In one season, and in one family consisting of twelve or fourteen persons, the following were the effects in as many individuals. One tertian; one double quotidian headach; another tertian; one diseased spleen; in one individual, aged only eighteen, a temporary hemiplegia with obscure quotidian; a second case of palsy in one leg in a person of twenty, with obscure quotidian and symptoms of diseased spleen: a regular Neuralgia of the face, of double tertian type. In a following, distant, season, and in some of the same persons, there occurred; palsy of the face with imperfect speech, an attack lasting beyond a week, and replaced by quotidian neuralgia (Tic); a double tertian, common intermittent, terminating in a quotidian, or double tertian, neuralgia; a quotidian with neuralgia in the shin bone; the same patient having had, in a preceding season, a common tertian so obscurely marked, that he was ordered to Italy for a consumption, (a consumption which was cured by two ounces of bark and a change of place to ten miles distance,) and, in a following one, having been attacked again with a double tertian, of which one fit was attended by the neuralgia of the shin and the other by a headach.

"This particular instance, it will be seen, embraces a considerable number of varieties under the two heads of Intermittent and Neuralgia; while I might even have extended it, by adding what occurred in other seasons, in the same place; among which I might have enumerated an irregular intermittent with neuralgic palpitation of the heart; an acute hepatitis (probably dependent on the same cause); two instances of diseased spleen; one of Neuralgia, with obscure intermittent, in the foot; one of periodical toothach with double quotidian; one of periodical quotidian rheumatism in the arm: one of quotidian with irritability of the bladder: a second, of very severe neuralgia of the heart, replaced and cured by a common quotidian; and one of a periodical general chronic rheumatism, of a most defined type and quotidian character." 324.

Our author has not a doubt, nor have we, that the same cause acting on different persons, produced all these disorders—more especially as the same individuals suffered, in different and subsequent seasons, several of the above-mentioned forms of disorder. A remarkable instance is stated, in the person of a gentleman, with whom the author was intimately acquainted for nearly thirty years, during the greater part of which he was harrassed with the various forms of chronic intermittent.

"In mere fever, this patient experienced various remittents, together with tertian, double tertian, quotidian, and double quotidian, in different years; and, in the anomalous varieties, what may perhaps be referred to the Asthmatica, and to the Stranguriosa, and also what may possibly be the Nephralgica of Sauvages; together with the Emetica, the Hysterica, and the Soporosa, of the same arrangement. These intermittents also, at different times, were united with, or succeeded to, or were replaced by, periodical and marked general chronic rheumatism, periodical local rheumatism in a limb, and rheumatism of the face, with repeated slight attacks of the ophthalmia of one eye, attended by hemicrania. In simple Neuralgia, this patient also experienced that of the face, repeatedly, long relapses of pure hemicrania, clavus, that of the eye, or optic nerve, sciatica, and a similar affection in one radial nerve and in the anterior crural; as, on different occasions, he suffered quotidian intermittent toothach, and the most severe neuralgia of the heart which I have ever witnessed, recurring annually for many years, replacing, once a local periodical rheumatism, and more than once replaced and cured by a quotidian simple intermittent.

"This is probably a rare case of severity and multiplicity, as the case itself was remarkable for its inveterate duration; but I doubt not that it can be paralleled, if not equalled, by the experience of others. But it is plain that it can be so paralleled, only by taking the same views of these diseases as myself; since, under the present opinions, most of those would have been considered as independent disorders, accidentally meeting in a single subject." 329.

To these two branches of evidence, community of cause—co-existence and interchangeableness—our author adds another, the effects (whether good or bad) of remedies. Throughout the whole catalogue, it is to the same class or system of remedies that we must look for the cure—and these are all remedies acting on the constitution—a fact that strongly confirms the view which our author has taken. Even when the remedies are not medicines, as change of air, mental impressions, &c. the same argument holds good. They act on the whole system, and thereby relieve the local neuralgic disease. Again, the same system of treatment which is injurious in one of these diseases is injurious in all the rest of the class—and the *same kind* of evil consequences arises in all as in one.

"Whether it be an intermittent of the most ordinary character, whether it be an anomalous one, or should it be any of all the neuralgic diseases, however apparently local and simple, it is the constitution, or the system at large, which suffers from that improper treatment, and in the same manner; while as far as the local, or even the general disease may suffer, it is, in all, also, in the same manner; namely, that they become confirmed or aggravated, or acquire a tendency to recur when they would otherwise have terminated." 333.

The following is an abstract of all that precedes, and in the words of the author.

"The abstract in question is therefore the following, passing over the community of remittent and intermittent fevers, as an admitted fact. Intermittent fevers arise from Malaria, certainly, as principally, and from mere cold possibly; but are renewable by mere cold, when once they have existed.

"They are often attended by peculiar local symptoms producing the anomalous varieties, while, when the febrile state is slight or obscure, these local disorders appear to be the chief disease.

"Such local disorders are either affections of the nervous system, or of an inflammatory character, and they have been fully described.

"The same intermittent fevers, more or less distinct, are accompanied by all the Neuralgiæ that have been described, whether these consist of simple pain, or are attended by inflammation; and when the febrile state is slight or obscure, those local affections appear to form the chief disease.

"If intermittent fevers alternate with all the anomalous local symptoms or diseases, so do they with all the neuralgic diseases: and in such cases, the supervention of one is the removal of the other.

"Thus also, all those local diseases, including all the Neuralgiæ, alternate with each other; or the appearance of one form is the cure of a preceding one.

"Many of the Neuralgiæ will exist almost simultaneously, or else in alternating paroxysms; these having any of the types of intermittent.

"They also exist in alternating paroxysms with simple intermittent: or a particular doubled type will consist alternately of a paroxysm of pure fever and a paroxysm of Neuralgia.

"The same individual, under a persevering intermittent, will experience many of the anomalous forms of that disease, and also many of the neuralgic diseases, in alternation or succession, or else in union; and, in such cases, the type, and the hour of recurrence, will be the same for all the forms, even through a long course of years.

"Malaria will produce the neuralgic diseases directly, as, probably, will mere cold; but they are renewable by mere cold when once they have existed; and in these cases, though the intermittent fever is probably always present, it may be so slight as to be overlooked. In this, the first cause, Neuralgia, in all its forms, resembles intermittent: but it differs, inasmuch as it can be excited by direct injury of a nerve; a difference however which is of no moment as to the general identity, because we know of no means of thus injuring the entire nervous system so as to produce general intermittent.

"The same Malaria, in the same spot, acting on different individuals at the same time, will produce either intermittent or Neuralgia, and every form of each.

"Intermittent and Neuralgia, in all their forms, are cured by the same remedies, and injured by the same wrong treatment; and those remedies are constitutional ones, whether the diseases be local or general; while, very particularly, the local and the general diseases both, are cured by operations on the imagination.

"The conspicuously wrong treatment for all of these diseases, whether Neuralgiæ or intermittents, consists in the debilitating practice, as the right treatment is found in what is esteemed the reverse; and whatever be the disease, be it local or general, when that practice is pushed so far as to become injurious, the injury is always of the same character, affecting the entire nervous system." 337.

We must pass over a short chapter, (XII.) on certain consequences of intermittent and neuralgia, in order to devote more of our remaining space to—

### Chap. XIII.—On the Cure of Neuralgia in general.

If our author be right in considering neuralgia as a disease dependent on a constitutional cause, however prominent may be the local symptoms—in short, if it be a mode of intermittent fever, or fundamentally of the same nature, it is natural that the same system of treatment should be enjoined. To this he was led, more than twenty years ago, from theory, and is now confirmed in the propriety of the system by practice and observation. This plan of treatment has never failed him in recent cases, and has often succeeded in those which were of long standing. In this chapter, our author has been unavoidably led into considerable repetition, as the principles of cure, and even many of the individual remedies, have been broached or detailed in preceding chapters, more especially when treating of intermittent. It will not be necessary for us, however, to go much into the minutiæ of the treatment; since it was of infinitely more importance to connect the etiology and pathology of these varieties of disease, than to dwell on their management when once recognized.

The first remark, and it is a very important one, is this—that the neuralgiæ often disappear without medicines, by a spontaneous effort of the constitution—while they are also truly cured by circumstances that are not noticed, and to which credit is not given. This explains the reputation which has been gained by particular modes of cure, which were, in reality, either nugatory or injurious in themselves. Hence improper practices are continued from mistaken observation. Particular periods of life, as the climacteric in males, and cessation of the catamenia in females, often root out old and inveterate neuralgic affections, that had defied all remedies. The most frequent of the real, though little observed causes of cure, however, will be found in change of air, and of general habits of life—which, by the bye, is a direct remedy of great power, though often recommended to the patient when the practitioner is tired out with fruitless attendance. The effects of moral impressions are under-rated and ridiculed. A change of physicians, or the acquisition of a new and strong confidence in a new and reputed person, often effects a cure, where the remedies prescribed had little or nothing to do in the business.

> "Hence an actual benefit often derived from empirical remedies and empirics, or from physicians of popular if false reputation, or of peculiar, perhaps insolent or coarse manners: an influence extending widely over all the nervous disorders, of which so many occur from the general cause of disease which includes the subjects of this essay." 370.

This, in reality, is the cure by charms. This is the reason why quack medicines, the composition of which, being unknown, is more respected, effect cures, when the same medicines fail in ordinary prescription.

> "Hence that universal confidence in substances and formulæ and numbers and quantity, and hence especially that enormous consumption of Empirical remedies; compounds found in every pharmacopœia, but divested of all their virtues under this form, because separated from the mystery and the incantation. The physician who attempts to reason with his patient on the effects and utility of his remedies, pays a most unmerited compliment to human reason: and while he will fail to influence, he will not be very long in discovering that he will shortly have no patients to enlighten or to cure. With the loss of the mystery, the merit is at an end: and he who proves himself to be the true philosopher and physician, is precisely the man who will never be trusted." 371.

This is a melancholy picture, but we fear it is too true. It may account for the immense reputation of a living practitioner, who never reasons or says a civil word to his patients, but drives them from his presence, all having, and all knowing before hand, that they will have the same prescription or box of pills, whatever be the nature of the malady! Dr. M. relates a case of tic douloureux, which he had long treated in vain with arsenic and other remedies, but which instantaneously vanished before the solemn gibberish of an old woman, celebrated for the possession of a charm against tooth-ache.

We know that intermittents are sometimes cured by giving a powerful anodyne just before the expected paroxysm, which breaks the chain, and interrupts the morbid process. The same is sometimes done in neuralgia, and ought not to be neglected, though they are not the real remedies in this class of maladies.

"But the chief and the most energetic remedies in Neuralgia, be the form what it may, are the tonics; and of these, as in intermittent, the most efficacious are bark and arsenic. Each, in its class, may stand at the head of a list which it is fruitless to enumerate, since it is so well known to even every druggist; nor need I repeat what relates to the mode of using these, since it is precisely the same as in intermittent fever. That there is any one vegetable tonic more efficacious than bark, or differing in the mode of action, as far as we now know these remedies and their powers, I am inclined to doubt: but not to deny that such do exist, since I consider that we are very far from having exhausted the medicines of the vegetable kingdom; so far, indeed, as rather to be in an absolute infancy of knowledge on this subject."

"While with bark as the type, the physician may command the whole range of astringents, aromatics, and bitters, he is also bound to try one where another fails; since thus may it possibly be discovered, even that what is most efficacious in common intermittent may not be most so in the Neuralgiæ, differing as they do in respect to the local action in the latter. But as I can, on this, say nothing of any great value from my own experience, I must be satisfied with having pointed out the leading principle and the road to be followed; as I need also do no more than suggest those combinations, whether of these vegetable substances themselves, or of the same with narcotics, the occasionally superior value of which in intermittent is well known.

"If arsenic be admitted as the type of the metallic remedies, it is equally easy for the physician to command the whole range of these: so well known, that I could add nothing respecting their powers; while I much suspect that very fanciful values have often been attached to some of them, from that common mechanical system which looks more to variety of medicines than to a knowledge of diseases. Much has indeed been lately said respecting the especial value of the carbonate of iron (as it is generally called) in the common Neuralgia (Tic); while in reality it has been administered as a merely empirical remedy, and without system. In my own experience, I had resorted to it long before these recommendations, both in intermittent and Neuralgia; but without discovering that it possessed any collateral merit above arsenic, while far less generally efficacious as a remedy. But, on all these remedies, I shall be very glad to hear of the experience of others, since I have wanted both temptation and opportunity to do them justice. As to the value of arsenic compared to bark, I can only repeat what I said formerly, that I have found it more generally efficacious in Neuralgia, while it has appeared less so in intermittent: often acting almost like a charm on the pain, and even in cases of many years' duration. But on this also I am ready to be corrected; as I am satisfied that the experience of no one individual, even were it far greater than mine has been, is sufficient to decide on subjects of this nature." 377.

Dr. M. makes no distinction, as to treatment, in the different forms of the disease—with the exception of sciatica, in which he has not had much experience. A medical friend, residing in a district noted for this disease, informs our author that he has derived the most marked advantage from this remedy in numerous cases.

When the attacks of intermittent or neuralgia are either very irregular or of long standing, the power of medicine is very limited in breaking the chain of morbid action. A single blood-letting has often rendered a recent intermittent regular, though previously irregular; and Dr. M. suggests, but without having experience on the point, a similar experiment in irregular neuralgia, while he condemns the practice of repeated depletion. Mercury, pushed so as to affect the mouth, will sometimes render agues amenable to tonics, though previously rebellious. The same may be tried in the neuralgiæ, since, in both classes, the glandular viscera are often deranged, and the mercury acts beneficially in correcting such disorders. But as the greater number of cases which present themselves are now chronic, and, consequently inveterate, probably from the wrong treatment employed when they were recent, so the cures will be comparatively few, however judicious the remedies. It is not until the old cases shall have died off, and a generation of the same diseases has arisen under the improved practice, that a fair trial can be given to the latter.

One great cause of neuralgia becoming chronic, is the caprice or impatience of the afflicted. Anxious for a speedy cure, they are led away in succession by name after name, and recommendation after recommendation, the consequence of which is, that no steady system is pursued, and no cure effected. The work, half done by one, is reversed by another, till, at length, the patient is rendered sceptical as to the skill of the practitioner or the potency of medicine.

But the paramount object is to withdraw the patient, if possible, from the operation of the primary causes of the disease. On this account, the locality of his residence should be carefully examined, according to the rules which have been already laid down by the author in his Treatise on Malaria, and of which the reader will find ample analyses in this Journal. Without such removal from the sphere of the causes, no permanent cure need be expected. The dread of moisture should ever be in the patient's mind—he should remove to a dry, but not to a cold situation, since cold itself is an exciting cause. The change of scene and air resulting from travelling alone, would often effect the cure, both in agues and the neuralgiæ.

"What remains as to the general treatment, relates to diet. As in intermittent, whether recent or chronic, I have no hesitation in saying that the usual full diet of persons in health, with a rational use of wine, forms an essential aid to the cure; and that it has often proved a cure in itself, when used as replacing the opposite and pernicious system. But I shall not enlarge on this; as the evils arising from low diet are involved in those belonging to the debilitating practice on which, even after all that I have said, I must offer some additional remarks hereafter." 386.

Of the local remedies for Neuralgia we need say but little. Dr. M. like Dr. Heberden, found blisters to aggravate the pain, when placed near the nerve affected. What has been called a perpetual blister is still worse, as proving "almost always a positive aggravation, not only of the local disease itself, but of the general irritation and disorder of the system."

"The only local remedy from which I have really seen such advantageous effects as to induce me to recommend it, is the application of steam directed by the usual means of a pipe, to the affected part; while of course, the same reasoning applies, if in a minor degree, to fomentations and hot water. The value of these latter applications, indeed, in rheumatism of the face, in the rheumatic or neuralgic ophthalmia, and in sciatica, has long been known; if, from their too great simplicity, and their not being 'made up in the apothecary's shop,' they are less valued than they deserve. But while I consider the blast of steam as the most effective of all the modifications of this practice, I have often succeeded by means of it, in removing, almost instantaneously, a paroxysm of the severest Neuralgia of the face, and, occasionally, so as to put a stop, in the chronic disease, to an entire relapse, which, from all the patient's past experience, was expected to last some weeks." 391.

Cold applied to the part does sometimes give temporary, but never permanent relief. On the contrary, it generally exasperates the subsequent sufferings of the patient.

"Though I have already spoken of the use of narcotics, this is a more convenient place to point out one advantage to be derived from them; a fact which I purposely postponed, on account of its connection with the useful effects of hot water and steam. As a means of diminishing pain during the painful state, they are nearly useless, unless pushed to such an excess as to stupify the patient; in which case, it is probable, as I already insinuated, that their effects are injurious, while it is easy to comprehend how they ought to be so, by inducing, indirectly, that debility which so prolongs and aggravates all the Neuralgiæ. But when the acute state is past, they become useful, as tending to remove that soreness which remains after the chief pain has ceased, and also by reducing the general irritation which has been excited by it. Thus also they sometimes act usefully, even as local applications, at least to sensible parts; and it is probably on this principle chiefly, that they are of advantage in the neuralgic inflammation of the eye." 394.

Dr. M. next adverts to the lædentia, and satirises with no small degree of force, the once celebrated practice of dividing the nerve in neuralgia; but as that practice is now laid in the "Tomb of the Capulets," we need not trouble our readers or ourselves on that point. The use or rather abuse of excessive purgation is next denounced by our author, and not without reason. Low diet, of course, comes in for its share of censure, and, as far as neuralgia is concerned, we have no fault to find with our author's strictures. But when he comes to ridicule the plan of abstemious living in dyspeptic complaints, he goes beyond his depth, and proves to those who have infinitely more experience than himself, that he knows nothing about the matter. This is the misery of having a hobby-horse. A man hits upon one good idea or thing; but he is not content with making that idea or thing useful to the world—he must push it to extremes, and endeavour to make it the "universal good." Dr. Macculloch must be well aware that no medical journal has done him so much justice as ours; and that we have proclaimed his merits through every region of the earth, which "the rising or the setting sun surveys." He is too sensible not to know that our praise is the more valuable in proportion to the impartiality which we display towards his failings—at least what we consider his failings. The following case, which we shall give in Dr. M.'s own words, does not at all support his anathema against *abstinence* in dyspepsia, though it is brought forward by him as a "coup de grace" to that system.

"This unfortunate philosopher had been long subject to the usual dyspeptic and nervous symptoms of studious men, and was of a sallow and emaciated complexion; appearing, in familiar language, to be far more in want of additional blood than of its abstraction, while his disorder was continuously aggravated by a system of low diet, adopted on the same mistaken views. Passing every day with him, in company with an English physician, it was easy to watch that over which we had no control; as there would also have been no propriety in attempting to oppose 'the best advice in Paris.' Headach was, as usual, one of the occasional symptoms; and on one unfortunate day he was induced to send for his surgical friend, by whom he was immediately bled. The headache, on the following day, continued, or rather returned, as it had formerly done, but with increased confusion of thought; the pulse and all else indicating, to the English physicians in question, increase of general debility, and compelling us at length to offer advice, which was however opposed by the usual arguments. A second blood-letting of course took place; and the consequence was that he became, but only in the night, partially delirious; a result easily explained, in its very limitation. It was then determined in full consultation, that there was inflammation of the brain, to the exceeding surprise, not without remonstrances, of the two English physicians; and, consequently, with the addition of blisters, shaving the head, and ice, another blood-letting was ordered and practised. The delirium then increased, while the pulse became feeble enough, as might have been supposed, to have made any man reflect; but as this did not happen, or rather as the reflections took the opposite course, the practice was persevered in, and on the following day the patient died: leaving the physicians, doubtless, convinced, as usual, that he had not lost blood enough. Such is a French case; but it would be easy to give no small number of parallels from English practice; and should it make no impression at present, the day will come round again when its value as well as its nature will be understood." 403.

Doubtless there might be many cases collected on both sides of the channel, where sanguineous depletion has been carried too far—and where irritation is mistaken for inflammation. This is the great source of error. Where inflammation actually exists, there cannot be very much mischief done by taking away a little more blood than is necessary. But where the *neuroses* are treated as *phlogoses*, which was the case with the unfortunate gentleman in Paris, then indeed the havoc of constitution is tremendous, and life itself is often sacrificed. With the following specimen of our author's sarcastic strictures on physicians and physic, we shall close this article.

"It were well indeed if not only ruined constitutions, but even death itself, were not the frequent, the almost daily result of physic thus misapplied in all the analogous and parallel cases, as also in some others; the produce of a combination of system, fashion, and ignorance, which renders Physicians and physic the just terror at present of all those who can see and distinguish. It is difficult to speak without high indignation as well as horror, of what we thus daily witness: to suppress the former is impossible, when our own, perhaps dearest friends, have thus been destroyed: and well now, perhaps, will he decide, who, like Napoleon, resolves to exclude this art and its professors entirely; for, on the arithmetical average, he will assuredly be far on the side of security. It is but to open our eyes to see the truth of this every day; while if it is over the ruined health, or the life, of females that we shall most often have occasion to grieve, from the obvious reason that in them the nervous affections thus mistaken and maltreated, chiefly abound, or are chiefly brought before physicians, so has there been a rapid increase of the evil, from the numbers who, returning from a continental residence with the consequences of marsh fever which I have so often described, have been subjected to this, truly mortal as well as mistaken treatment." 421.

The last chapter in the work is one of a different cast from the others. Having terminated his Essay when his evidence was exhausted, and his induction carried as far as it could safely go, Dr. M. ventures on a chapter of "conjectures respecting the condition of the nerves and nervous system in intermittent and neuralgic diseases." These conjectures are ingenious, and some of them plausible; but we have no space left for samples of them here. We part from our author with feelings of much respect and esteem—believing that he has contributed much more to the advancement of our science than many who have held their heads much higher in the republic of medicine.

